# Ubiquitin Carboxyl-Terminal Hydrolases and Human Malignancies: The Novel Prognostic and Therapeutic Implications for Head and Neck Cancer

**DOI:** 10.3389/fonc.2020.592501

**Published:** 2021-01-29

**Authors:** Chao Rong, Ran Zhou, Shan Wan, Dan Su, Shou-Li Wang, Jochen Hess

**Affiliations:** ^1^ Department of Pathology, School of Biology & Basic Medical Sciences, Soochow University, Suzhou, China; ^2^ Section Experimental and Translational Head and Neck Oncology, Department of Otolaryngology, Head and Neck Surgery, University Hospital Heidelberg, Heidelberg, Germany; ^3^ Department of Pharmacy, The First Affiliated Hospital of University of Science and Technology of China (USTC), Division of Life Sciences and Medicine, University of Science and Technology of China, Hefei, China; ^4^ Research Group Molecular Mechanisms of Head and Neck Tumors, Deutsches Krebsforschungszentrum (DKFZ), Heidelberg, Germany

**Keywords:** head and neck cancer, ubiquitin C-terminal hydrolases, deubiquitinating enzymes, genomic alteration, clinical relevance

## Abstract

Ubiquitin C-terminal hydrolases (UCHs), a subfamily of deubiquitinating enzymes (DUBs), have been found in a variety of tumor entities and play distinct roles in the pathogenesis and development of various cancers including head and neck cancer (HNC). HNC is a heterogeneous disease arising from the mucosal epithelia of the upper aerodigestive tract, including different anatomic sites, distinct histopathologic types, as well as human papillomavirus (HPV)-positive and negative subgroups. Despite advances in multi-disciplinary treatment for HNC, the long-term survival rate of patients with HNC remains low. Emerging evidence has revealed the members of UCHs are associated with the pathogenesis and clinical prognosis of HNC, which highlights the prognostic and therapeutic implications of UCHs for patients with HNC. In this review, we summarize the physiological and pathological functions of the UCHs family, which provides enlightenment of potential mechanisms of UCHs family in HNC pathogenesis and highlights the potential consideration of UCHs as attractive drug targets.

## Introduction

Head and neck cancer (HNC) represents the seventh most prevalent human malignancies with an annual incidence of 890,000 new cases worldwide, including 76,000 cases in China and 18,260 cases in Germany (1, 2). Anatomically, HNC occurs at distinct sites including lip, oral cavity, nasal cavity, sinonasal cavity, nasopharynx, oropharynx, hypopharynx, larynx, and salivary glands, and etiologic risk factors, epidemiology, treatment strategies as well as clinical outcome differ among individual subsites (3). Over 90% of cases are diagnosed as head and neck squamous cell carcinoma (HNSCC), which arises from the mucosal epithelia of the upper aerodigestive tract. High incidence areas for oral cavity cancer include Middle and South Asia, Western and Southern Europe as well as South Africa. The incidence of oropharyngeal SCC (OPSCC) is elevated in Europe and North America. Nasopharyngeal cancer (NPC) is most common in East and Southeast Asia, especially in South China (4). Tobacco smoking and heavy alcohol consumption have been identified as the most important risk factors in developed countries (5). In developing countries, risk factors also include EpsteinBarr virus (EBV) infection for NPC, areca nut chewing, consumption of preserved foods, and oral hygiene (6–8). During the past two decades, the overall incidence of HNSCC has gradually decreased in western developed countries. However, a subgroup of HNSCC, particularly OPSCC, has been becoming more prevalent in young adults, which is attributed to high-risk human papillomavirus (HPV) infection, predominantly HPV16 (9). High-risk HPV types comprise two oncogenes, E6 and E7, which inactivate the tumor suppressors p53 and retinoblastoma (RB), respectively. As a result, cell cycle progression and cell death in infected cells are disrupted, as initial steps for HPV-related carcinogenesis (10–13). Besides the viral oncogenes E6/E7, HPV E2, E4, and E5 have been shown to facilitate the synergistic effects of viral oncogenesis, which represents an alternative manner to HPV-induced carcinogenesis (14). It has been well-established that HPV-positive and HPV-negative OPSCC have distinct differences in gene expression profiles, genomic alterations, immune landscape, as well as clinical outcomes (15–18). Due to the more favorable prognosis of HPV-positive OPSCC, clinical trials have been launched to investigate HPV-stratified de-escalation treatment based on currently established protocols. However, final results and definitive conclusions are pending, which might improve the post-treatment quality of HPV-related OPSCC patients (19–22).

Despite advances in multi-disciplinary treatment for HNSCC, including surgical approaches, radiotherapy, chemotherapy, molecular-targeted therapy, and immunotherapy, the overall survival of advanced HNSCC has only improved slightly, and appropriate therapy remains a major challenge. Over the past decade, large-scale genomic profiling and proteomic studies, including The Cancer Genome Atlas (TCGA) projects, have highlighted a comprehensive molecular landscape of changes in DNA copy number, somatic mutations, promoter methylation, and protein and gene expression, indicating the critical components and signal pathway in HNSCC pathogenesis (23–25). A better understanding of these molecular underpinnings may inspire novel drug targets as well as molecular biomarkers for personalized treatment.

The proteome is exceedingly complex and has been regarded as the major driver or actuator of fundamental cellular processes. Protein ubiquitination is a post-translational modification process that plays critical roles in numerous biological processes, including cell growth and differentiation, signal transduction, DNA repair, and oncogenesis (26). The conjugation of ubiquitin (Ub) to target proteins is catalyzed by a cascade of ubiquitinating enzymes, including Ub-activating enzymes (E1s), Ub-conjugating enzymes (E2s), and Ub ligases (E3s). Conjugation of Ub to a substrate lysine, its lysines or its N-terminus, results in the generation of different substrate ubiquitin structures, which can be either a mono- or poly-ubiquitylation process and allows targeted proteins to fulfill a diverse range of functions. However, protein ubiquitination is highly reversible. Deubiquitinases or deubiquitinating enzymes (DUBs) catalyze the removal of ubiquitin from target proteins to generate free monomeric Ub (27). The human genome encodes approximately 100 DUBs categorized into six subfamilies: the ubiquitin C-terminal hydrolases (UCHs), the ubiquitin-specific proteases (USPs), the ovarian tumor proteases (OTUs), the Josephin or Machado-Joseph disease protein domain proteases (MJDs), the Jab1/MPN domain-associated metalloisopeptidase (JAMM), and the monocyte chemotactic protein-induced protein family (MCPIP) (27). Among these families, UBPs are mostly described to date, with 60 proteases in humans, which have been well-reviewed by a range of publications (28–31). Recent studies have revealed the emerging functions of UCHs in the pathogenesis and progression of human malignancies. However, few studies on UCHs in HNC are available. One member of UCHs family, BRCA1-associated protein-1 (BAP1), was identified to be associated with poor outcome following radiation in HPV-negative HNSCC clinical sample by proteomic and transcriptomic analysis (32). Moreover, another member of UCHs family, UCHL1 was demonstrated as a tumor suppressor gene in nasopharyngeal carcinoma (NPC) (33). In this review, we systematically summarize the physiological and pathological functions of the UCHs family in human malignancies, providing enlightenment on potential mechanisms of UCHs family in HNC pathogenesis and the potential consideration of UCHs as novel promising drug targets.

## Structures and Functions of UCHs

**Figure 1:**
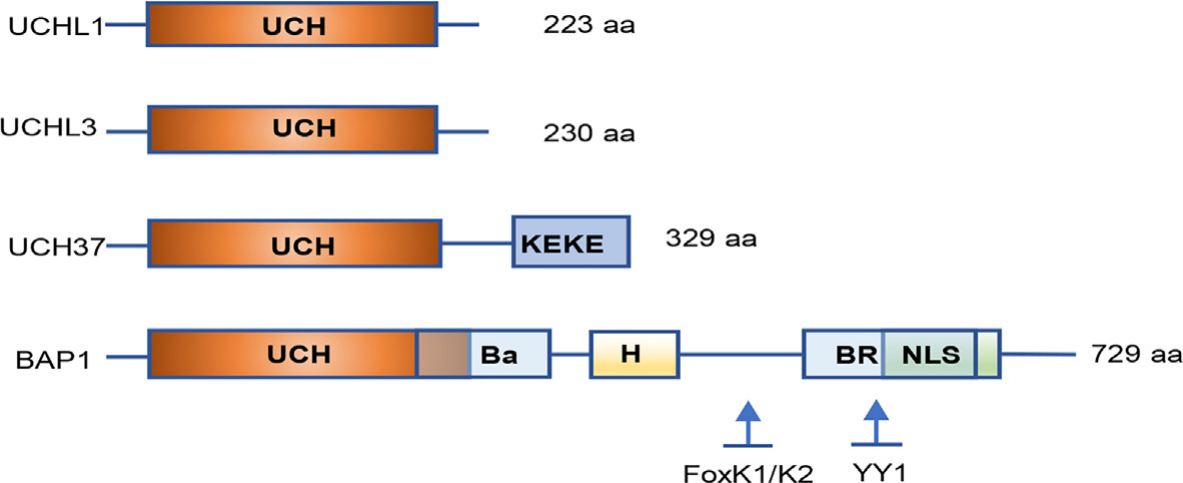
Simplified structure of the UCH family proteins. All UCH members share close homology in their catalytic domains and have a core catalytic domain with 230 amino acids. UCHL3 contains a KEKE motif in the C-terminal tail. BAP1 consists of a long C-terminal extension illustrating numerous functional domains and binding sites for interacting proteins. UCH, ubiquitin C-terminal hydrolase; Ba, BARD1 binding domain; H, HCF-binding motif; BR, BRCA1 binding domain; NLS, nuclear localization signal; YY1, Ying Yang 1 binding region. Inspired by (26).

Among DUBs family, molecular structures of UCHs were the first to be characterized. Four UCHs in humans have been identified: UCHL1/PGP9.5 (protein gene product 9.5), UCHL3, UCHL5/UCH37, and BAP1. All UCHs share a core catalytic domain with 230 amino acids and close homology among family members. They comprise a confined loop that cleaves short ubiquitylated peptides (up to 20–30 amino acids) from the C-terminal glycine residue (**Figure 1**).

### UCHL1

First identified as a member of UCHs family in the 1980s, UCHL1 is an abundant neuronal protein containing only one UCH domain with very short N- and C- terminal extensions. It possesses one of the most complicated protein knotted structure, which is regarded to protect UCHs from degradation in the proteasome as well as to maintain proper proteasomal function. Besides its DUB function in the recycling of free Ub, UCHL1 is also known to have a ubiquitin ligase activity as a mono-Ub stabilizer by preventing its degradation (34). It was reported that the generation rate of monomeric Ub by UCHL1 *in vitro* is enhanced by the catalytic residues C90 and H161 (35). Analysis of the crystal structure suggests that UCHL1 preferentially binds monomeric or small adducts of Ub, but does not act on large polymers of Ub (36). Therefore, UCHL1 has the potential for numerous ubiquitination-dependent biological processes.

UCHL1 is predominantly expressed in the brain, where it comprises up to 5% of total neuronal proteins. Although the precise function of UCHL1 is not fully clarified in many pathological processes, better understandings of functional UCHL1 has been largely reported in neuronal dysfunction and neurodegenerative disorders (34, 37–39). For instance, the specific distribution and activity of UCHL1 in human tissues has the potential clinical significance for Parkinson’s disease (PD) and Alzheimer’s disease (AD), might be a major target of reactive oxygen species (ROS) damage (40). Although most cases of PD are sporadic, a small subgroup of PD has been linked to specific genomic mutations (41). Interestingly, The I93M point mutation in UCHL1 has been reported to be associated with PD susceptibility by decreasing hydrolytic activity (42). By contrast, an S18Y variation in UCHL1 shows a protective enzyme with a reduced risk of PD by a reduction of α-synuclein (43). A study has shown that modification of the UCHL1 C152 site decreases injury to gray and white matter, resulting in the recovery of motor function after middle cerebral artery occlusion (44). Another potential feature of UCHL1 is an ATP-independent E3 ligase activity, which promotes Lys63 (K63) polyubiquitination of α-synuclein (34). Moreover, UCHL1 was demonstrated as a novel interactor and substrate of PD linked E3 ubiquitin-protein ligase parkin by the autophagy-lysosome pathway (45).

It has also been reported at much lower levels in kidney, breast epithelium, and reproductive tissues (46, 47), and to be expressed context-dependent in individual cells, such as human fibroblasts during wound healing (48). Uniform cytoplasmic staining of UCHL1 was observed in neurons, but UCHL1 can translocate into the nucleus and regulate microtubule dynamics (49). Although it is absent in most other normal tissues, UCHL1 appears to be aberrantly expressed in many non-neuronal tumors, including breast, prostate, colorectal, gastric, head and neck, and pancreatic ductal carcinomas (33, 50–55). The functions and potential mechanisms of UCHL1 in tumorigenesis have been reviewed by several excellent publications (56, 57). The interactive proteins with UCHL1 as well as other UCH family members in human malignancies are summarized in **Table 1**. In addition, altered expression levels of UCHs in various cancers have also been reviewed in **Table 2**.

**Table 1:** Overview of interacting proteins with UCHs family in various human malignancies.

UCHs family	Interacting protein	Human malignancies	References
UCHL1	P53	Breast cancer, metastatic colon adenocarcinoma, nasopharyngeal carcinoma, hepatocellular carcinoma	(33, 58–60)
	MDM2	Colorectal cancer, prostate cancer, nasopharyngeal carcinoma	(33, 61)
	β-catenin	Colorectal cancer, pediatric high-grade glioma	(62, 63)
	NOX4	Cervical cancer	(64)
	EGFR	Breast cancer	(65)
	HIF-1	Breast, lung cancer	(66, 67)
	cyclin B1	Uterine serous cancer	(68)
	PHLPP1	Lymphoma, lung tumor	(69)
	MITF	Melanoma	(70)
	SMAD2	Breast cancer	(53)
	TGFβ type I receptor	Breast cancer	(53)
	CTTN	Nasopharyngeal carcinoma	(71)
	mTORC1	B-cell lymphoma	(72)
	eIF4F	B-cell lymphoma	(73)
UCHL3	TRAF2	Ovarian cancer	(74)
	BRCA2	Breast cancer	(75)
	RAD51	Breast cancer	(75)
UCH37/UCHL5	PRP19	Hepatocellular carcinoma	(76)
	PRDX1	Hepatocellular carcinoma	(77)
	SNRPF	Glioma	(78)
	Smad2	Ovarian cancer	(79)
	GRP78	Hepatocellular carcinoma	(80)
	E2F1	Liver cancer	(81)
	Rpn13	Cervical cancer	(82)
BAP1	BRCA1/BARD1	Breast cancer, chronic myeloid leukemia, meningioma	(83–85)
	HCF-1	Breast cancer, renal cell carcinoma	(86, 87)
	Ino80	Mesothelioma	(88)
	Gamma-tubulin	Breast cancer	(89)
	ASXL1/2	Mesothelioma	(90)
	MCRS1	Renal cell carcinoma	(91)
	Histone H2A	Head and neck cancer	(32)
	IP3R3	Prostate cancer	(92)
	ATF3	Multiple carcinomas	(93)
	14-3-3 protein	Neuroblastoma	(94)
	SLC7A11	Multiple carcinomas	(95, 96)

**Table 2:** Expression regulation of UCHs family in human malignancies.

UCHs family	Human malignances	Possible variations (References)
UCHL1	Breast cancer	Down-regulation (58)
	Hepatocellular carcinoma	Down-regulation (59)
	Invasive and metastatic breast cancer	Up-regulation (53, 97)
	Metastatic colon adenocarcinoma	Up-regulation (62)
	Nasopharyngeal carcinoma	Down-regulation (33, 71)
	Prostate cancer	Down-regulation (61)
	Pediatric high-grade glioma	Up-regulation (63)
	Ovarian cancer	Down-regulation (98)
	Non-small cell lung cancer	Up-regulation (99)
	Uterine serous cancer	Up-regulation (68)
	B-cell lymphoma	Up-regulation (100)
UCHL3	Breast cancer	Up-regulation (50)
	Ovarian cancer	Up-regulation (74)
	Non-small cell lung cancer	Up-regulation (101)
	Metastatic prostate cancer	Down-regulation (102)
	Cervical cancer	Up-regulation (103)
UCH37/UCHL5	Hepatocellular carcinoma	Up-regulation (76)
	Glioma	Down-regulation (78)
	Cervical cancer	Up-regulation (103)
	Esophageal squamous cell carcinoma	Up-regulation (104, 105)
BAP1	Breast cancer	Down-regulation (83, 89)
	Chronic myeloid leukemia	Down-regulation (84)
	Mesothelioma	Down-regulation (85, 106, 107)
	Non-small cell lung cancer	Down-regulation (83)
	Renal cell carcinoma	Down-regulation (87, 108)
	Uveal melanoma	Down-regulation (109, 110)
	Basal cell carcinomas	Down-regulation (111)
	Neuroblastoma	Down-regulation (94)
	Esophageal squamous cell carcinoma	Down-regulation (112)

Recent findings have revealed significant functions for UCHL1 in immune response and regulation. UCHL1 was found in mouse kidney, spleen, and bone marrow-derived dendritic cells, and its expression and activity were strongly regulated by the immune stimuli LPS and IFN-*γ* (113). UCHL1 modulates antigen processing by affecting the colocalization of intracellular MHC I with late endosomal/lysosomal compartments necessary for cross priming of CD8 T cells (113). Interestingly, an induced UCHL1 expression was also demonstrated in multipotent mesenchymal stromal cells (MSCs) upon stimulation with proinflammatory cytokines IFN-γ plus TNF-α, and negatively regulated the immunosuppressive capacity and survival of MSC. This discovery may provide potential MSC-based immunotherapy for inflammatory diseases by modulation of UCHL1 (114).

### UCHL3

UCHL3 and UCHL1 have significant structural similarity. However, the biological characteristics of UCHL3 are quite distinct concerning expression patterns and ligase activity. Unlike UCHL1, which is mainly restricted to neuronal and neurosecretory tissues, UCHL3 is more widely expressed throughout mammalian tissues. Interestingly, UCHL3 hardly exhibits ligase activity, while its hydrolytic activity is two-hundred-fold higher than UCHL1 toward a fluorogenic ubiquitin C-terminal amide (34). It was reported that UCHL3 enables to cleave the C-terminus of NEDD8, which is a ubiquitin-like protein that exerts the function of Ub to be conjugated to a lysine residue of the substrate (115). Next, UCHL3 has also been demonstrated to alleviate cryptorchid-induced germ cell apoptosis in *gad* mice. UCHL3 appears to have dual affinities for ubiquitin and Nedd8, and function as a deNEDDylating enzyme *in vivo*, suggesting that UCHL3 plays a critical role in germ cell apoptosis (116). Several studies using similar UCHL3 knockout mouse models revealed the significant functions in photoreceptor cell degeneration, neurodegeneration, fertilization and embryogenesis, stress responses in skeletal muscle, diet-induced obesity, and osteoblast differentiation (117–122). It is worth mentioning that level of UCHL3 protein in several neurodegenerative diseases is unchanged, while it hydrolyzes the C-terminal extension of a mutant ubiquitin (UBB^+1^), contributing to the role in neurodegenerative disorders (123).

An increasing number of studies have demonstrated vital functions of UCHL3 on tumorigenesis, including breast, prostate, ovarian, and non-small cell lung cancer (**Table 2**) (50, 74, 101, 102). Luo et al. found that UCHL3 deubiquitinates RAD51 and subsequently facilitates RAD51-BRCA2 interaction, which is critical for homologous recombination (HR) and contributes to therapeutic resistance in breast cancer (75). By contrast, UCHL3 is reduced in metastatic prostate cancer cell lines, and knockdown of UCHL3 promotes epithelial-to-mesenchymal transition (EMT), contributing to cancer cell invasion and metastasis (102). In contrast, high UCHL3 expression was reported in ovarian cancer and predicted a worse clinical outcome. The elevated UCHL3 facilitates carcinogenesis and enhances inflammation by deubiquitinating and stabilizing TNF Receptor Associated Factor 2 (TRAF2) (74). Taken together, the UCHL3 function in cancer remains controversial, suggesting the roles of UCHL3 is complicated and context-dependent in individual tumor types.

### UCH37

UCH37 (also known as UCHL5) was identified first as a 19S-associated deubiquitinating enzyme in the 1990s, which comprises a C-terminal extension (residues 227-329) in addition to an N-terminal UCH domain (residues 1–226) (124). It is specific for the distal subunit of Lys48-linked poly-Ub chains. Isolated full-length UCH37 displays weak catalytic activity due to autonomic inhibition by the C-terminal extension (125). The proteolytic activity requires a Ub receptor called ADRM1 (named hRpn13 in humans) binding to UCH37 *via* its C-terminal 46 residues (also called the KEKE motif) (125). In addition, hRpn13 was found to directly enhance the de-ubiquitination activity of UCH37 *in vitro* (125–127). The hRpn13-UCH37 complex hydrolyzes large Ub conjugates with incorporation into the 19S complex. By contrast, UCH37 is inhibited by the chromatin remodeling complex component INO80G mediated by the N-terminal domain of NFRKB (nuclear factor related to κB, NFRKB) (128). Rpn13 and INO80G share a conserved deubiquitinase adaptor (DEUBAD) domain that interacts with the C-terminal of UCH37, revealing conformational plasticity to regulate deubiquitinating activity on or off, respectively (128). Functionally, UCH37 is reported to perform a crucial role in certain protein-protein interactions involving several physiological and pathological processes, including development, cell proliferation, and apoptosis, hippocampal synaptic plasticity, Alzheimer’s disease, pulmonary fibrosis, as well as human malignancies (26, 129–133).

Wicks and colleagues reported UCH37 interacts with Smad7 to control TGF-β/Smad signaling activity, suggesting that UCH37-mediated deubiquitination might contribute to tumorigenesis (134). The first direct evidence of UCH37 in cancer study was described by a chemistry-based functional proteomics approach in cervical carcinoma. Activity profiling showed UCH37 is induced in the majority of carcinoma tissues and HPV E6/E7 immortalized human keratinocytes, indicating a significant role of UCH37 in tumor transformation (103). Subsequently, an increasing number of studies reported the potential functions in tumor cell proliferation, apoptosis, migration, and invasion, as well as clinical implications (**Table 2**) (76–78, 135–138).

### BAP1

The BAP1 protein consists of 729 amino acids that are encoded by the *BAP1* gene located on human chromosome 3p21.1. BAP1 protein was identified as a nuclear-localized DUB. In addition to the N-terminal UCH domain, BAP1 comprises a long C-terminal extension (**Figure 1**). BAP1 was originally found to interact with the RING finger domain of BRCA1 and to perform the cell growth-suppressive function. BAP1 is also involved in chromatin modification and transcription by deubiquitinating lysine residues in HCF1 and YY1. Both recruit histone-modifying complexes and regulate expression of numerous genes involved in multiple physiological processes (139). Moreover, BAP1 interacts with the transcription factor FOXK1/K2 in a phosphorylation-dependent manner, which represses FOXK2-target genes forming a ternary protein complex in which BAP1 bridges FoxK2 and HCF-1. Loss of BAP1 causes the increase of FoxK2 target genes, which is dependent on the Ring1B-Bmi1 complex (140).

Polycomb group proteins exert critical roles in transcriptional regulation, which contributes to a variety of physiological processes, including embryonic development, differentiation, and self-renewal. Polycomb repressive complexes (PRCs) are responsible for histone ubiquitination and methylation (139). BAP1 interacts with additional sex combs like 1 (ASXL1), forming a polycomb group repressive deubiquitinase complex (PR-DUB). The transcriptional function is regulated through histones modification *via* ubiquitination by PRCs and deubiquitination by PR-DUB. Thus BAP1 deficiency significantly alters ubiquitination level of histone 2A, leading to the dysregulation of cell cycle and cellular senescence (141). A recent study found cytoplasm BAP1 localizes at the ER, where it regulates type 3 inositol-1,4,5-trisphosphate receptor (IP3R3), modulating calcium (Ca^2+^) release from the endoplasmic reticulum into the cytosol and mitochondria, promoting apoptosis, which plays a critical role in cellular transformation (92). Another study has identified cystine transporter SLC7A11 as a critical BAP1 target gene in human malignancies, which was repressed by BAP1, causing increasing lipid peroxidation and ferroptosis (95).

BAP1 functions as a tumor suppressor through chromatin modulation, transcriptional regulation, cell cycle control, cellular differentiation, and DNA damage repair (142). Loss or mutation of *BAP1* gene is a common event in cancer and serves as a potential pathogenetic mechanism in various human malignancies, including uveal melanoma, mesothelioma, small cell and non-small cell lung carcinomas, renal cell carcinoma (RCC), breast cancer, and hepatocellular carcinoma (**Table 2**) (107, 143–148). Tumors associated with BAP1 somatic mutations have already been discussed in recent reviews (139, 149). Other alterations in the BAP1 gene have been reported, such as large deletions of exons causing premature protein termination, frameshift mutation, splice site mutations, and base substitutions-induced nonsense and missense mutations (143, 149). BAP1 acts as a tumor suppressor depending on both deubiquitination activity interfered by missense mutations and loss of nuclear localization signal by truncating mutations. Furthermore, several studies showed that BAP1 loss or modification is associated with different tumor phenotypes and clinical outcomes (108, 110, 150–152). For example, BAP1-mutated mesothelioma is significantly correlated with female predominance, younger age at onset, epithelioid differentiation, and better prognosis (153). At the same time, BAP1 mutation is strongly associated with a more aggressive, metastatic phenotype in uveal melanomas (143). BAP1 is frequently mutated in sporadic clear cell RCC with an incidence rate of 6–17%, which is associated with high tumor grade, rhabdoid/sarcomatoid transformation, and poor clinical outcome (154, 155). From a therapeutic standpoint in renal cell carcinoma, inactivation of BAP1 sensitizes tumor cells to irradiation and PARP-inhibitors, which might be due to the impaired ability of double-stranded DNA breaks (87).

## UCHs Members in HNC

Although UCHs members have been well investigated in a variety of human malignancies, the exact function of these enzymes in HNSCC pathogenesis and progression remain elusive. Each member of the UCHs family exerts distinct roles depending on the various tumor types. For example, UCHL1 has been controversially considered as a tumor suppressor or tumor promoter in specific tumor types. It was reported that UCHL1 is silenced by promoter CpG hypermethylation in a large panel of primary tumors including HNSCC cell lines and primary tumors, suggesting a tumor-suppressive function (33, 156). The methylation of the CpG locus associated with the UCHL1 gene is dependent on the anatomic site of HNSCC primary tumors, with most hypermethylation of UCHL1 specifically in oral cavity SCC (157). Restored UCHL1 expression significantly suppressed tumor cell proliferation and induced cellular apoptosis through activation of the p14ARF-p53 signaling pathway (33). A more recent study in nasopharyngeal carcinoma revealed a similar conclusion that UCHL1 promoter hypermethylation was validated in nasopharyngeal carcinoma tissues. In addition, restoration of UCHL1 inhibits tumor invasion and metastasis *in vitro* and *in vivo*. UCHL1 exerts tumor suppressor function by inducing K48-linked ubiquitination of CTTN (71). Currently, it is widely accepted that high-risk HPV infection is a risk factor for HNSCC, particularly in the oropharynx. High-risk HPV infects the oropharyngeal epithelium causing host immune suppression and evasion (11). UCHL1 does not assist HPV genome replication and viral propagation, but suppresses keratinocyte-mediated production of inflammatory cytokines and chemokines, thereby contributing to immune evasion and HPV persistent infection (158). UCHL1 interacts with tumor necrosis factor receptor-associated factor 3 (TRAF3), which acts as a negative regulator of the alternative NF-κB pathway and antiviral type I IFN activation. TRAF3 has been shown as a tumor suppressor that regulates the malignant phenotype of HPV-positive HNSCC (158).

As a tumor suppressor, BAP1 is critical for promoting DNA repair and cellular recovery from DSB *via* modulation of H2A ubiquitination (159). BAP1 was found to mediate radioresistance in an *in vivo* xenograft model and HNSCC cell lines *via* the deubiquitination of H2A and modulation of HR. Moreover, up-regulation of BAP1 was associated with worse clinical outcome in HNSCC, which indicates BAP1 might serve as a potential therapeutic target in HNSCC (32). In summary, it seems that loss of BAP1 foster genomic instability in tumor pathogenesis, however, the activity of BAP1 promotes tumor cell survival and contributes to therapeutic resistance during irradiation.

Induced activity of UCHL1 and UCHL3 were observed in E6/E7 immortalized primary keratinocytes, indicating the potential function of UCHL1 and UCHL3 in HPV-related HNSCC (103). However, few direct evidences concerning the function of UCHL3 and UCH37 in HNSCC have been reported.

**Figure 2:**
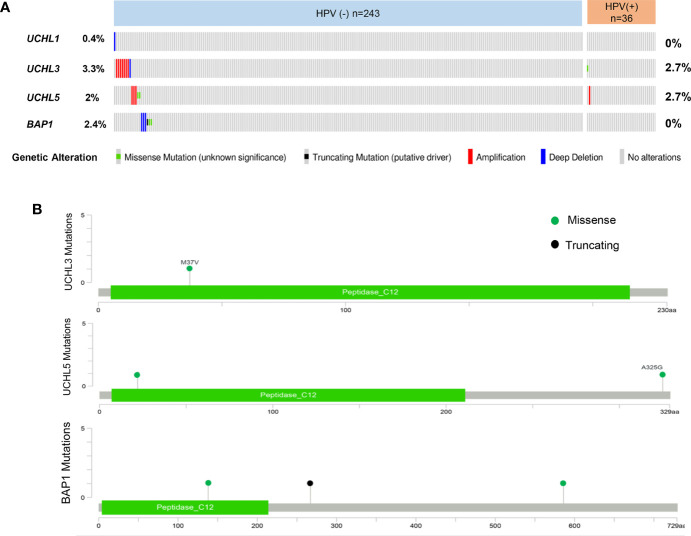
Overview of genetic changes of UCHs family in TCGA HNSCC patients. **(A)** Oncoprint shows altered UCHs family genes. The colors are associated with one class of variants, and the percentage (%) of patients affected is shown on the graph. **(B)** cBioPortal predicted mutation maps showing the positions of mutations on the functional domains of UCHL3, UCHL5, and BAP1 proteins.

Recently, comprehensive epigenetic and genomic profiling studies have highlighted the most frequently altered genes and signaling pathways in HNSCC. The genomic characterization of 279 HNSCCs including HPV-positive and HPV-negative tumors, has been published (23). Moreover, the molecular profiling data from over 500 HNSCC patients are available at the cBioPortal for Cancer Genomics, which provides interactive exploration and analysis of genetic alterations (160, 161). In addition, the GTEx project provides RNA sequencing data from more than 8,000 normal tissues. Currently, several web-based tools deliver interface-friendly and personalized functions based on TCGA and GTEx data (161, 162). cBioPortal provides visualization for the genomic alteration data. Clinical and genomic analysis of multicohort HNSCC has demonstrated that HPV-positive and HPV-negative tumors present heterogeneity in anatomical regions, mutation profiles, molecular characteristics, immune landscapes, and clinical prognosis. Many evidence revealed the diversity and heterogeneity of HNSCC clinicopathology and therapeutic responses depending on HPV status (163). To better understand the UCHs family mutational landscape in HNSCC, the cBioPortal tool was used to display the types of mutations and their positions in the domain structure of proteins (**Figures 2A, B**). UCHs member genes are altered in 22 (8%) of queried patients. Of these, 20 cases are HPV-negative, and 2 cases are HPV-positive.

**Figure 3:**
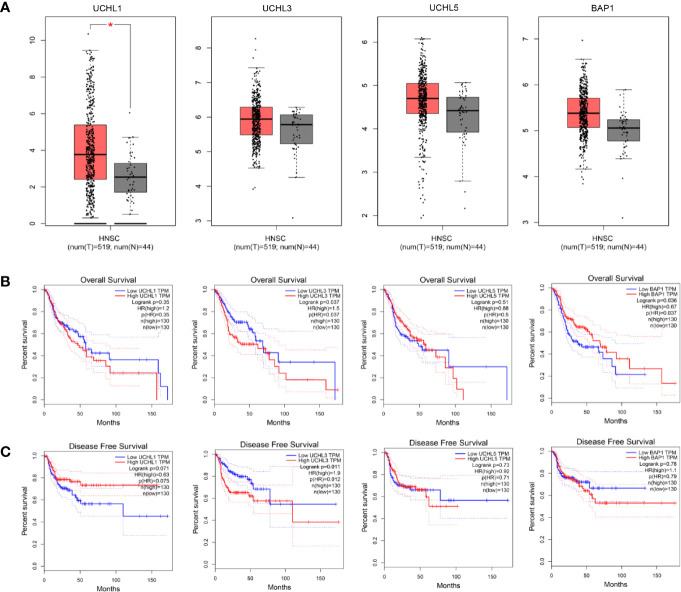
Differential expression analysis of UCH family genes between tumor tissues and normal tissues in the TCGA-HNSC cohort **(A)**. Kaplan-Meier plots analyses show overall **(B)** and disease-free **(C)** survival compared by log-rank test. The Cox proportional hazard ratio (HR) is shown in the survival plots.

*UCHL1* alterations accounted for 0.4% in HPV-negative subgroup and no genetic alteration in HPV-positive patients, *UCHL3* for 3.3% in HPV-negative and 2.7% in HPV-positive, *UCHL5* for 2% in HPV-negative and 2.7% in HPV-positive, and *BAP1* for 2.4% in HPV-negative and 0 in HPV-positive. Interestingly, there is no samples overlapped. Concerning the mutation type, one missense mutation in UCH-domain of *UCHL3*, two missense mutations in *UCHL5*, two missense mutations and one truncating mutation in *BAP1*. A web-based tool GEPIA (164) analysis revealed *UCHL1* gene expression in HNSCC tissues is significantly elevated as compared to normal tissues, which is different from the previous studies in nasopharyngeal carcinoma (71) (**Figure 3A**). Survival analyses based on gene expression levels was also applied to evaluate the clinical relevance of UCHs family genes (**Figures 3B, C**). The quartile cut-off method was determined depending on the optimization and visualization of the online web tool. However, numerous problems remain unsolved. We were not able to divide the cohort into two subtypes due to the incompleteness of the HPV status information. More specific subgroups of HNSCC patients for certain phenotypes need to be discovered depending on the protein expression patterns of UCHs family, which may contribute to illuminate the clinical relevance of UCHs family for HNSCC patients. Moreover, the gene networks regulated by UCHs family genes should be identified by analyzing the RNA-sequencing profiling data. Novel signaling pathways and biological processes related to UCHs family in HNSCC are urgent to be clarified. Functional proteomics represents a useful approach to investigate the UCHs family activity-related biological processes in different subtypes of HNSCC. Only BAP1 protein expression data by reverse-phase protein arrays (RPPAs) are available in the TCPA dataset, where BAP1 serves as a strong prognostic predictor for female-related cancer cohorts including samples of invasive breast carcinoma, Ovarian serous cystadenocarcinoma, Uterine Corpus Endometrial Carcinoma (25). More large-scale proteomic profiling data on the other UCHs members are urgent to be produced.

### Therapeutic Implications for HNC Targeting UCHs members

Research on targeting UCHs members in HNSCC therapy is still in the initial period. To our knowledge, there are no studies focusing on UCHs family molecular inhibitors or drugs for clinical trials, which reflects the lack of theoretical and preclinical research. Encouragingly, UCHs family has been shown to predict therapeutic sensitivity and clinical outcomes for various tumors. For example, UCHL1 strengthens tumor cells chemosensitivity in melanoma and colorectal cancer by stabilizing NOXA (165). BAP1 was also reported to modulate cancer cell sensitivity to radiotherapy and the molecular inhibitors including PARP (olaparib) or histone deacetylase inhibitors (panobinostat), which may become potential therapeutic strategies (87, 166). The small molecule b-AP15 as a previously unidentified class of proteasome inhibitor abrogates the activity of two 19S regulatory-particle-associated deubiquitinases, UCH37/UCHL5, and USP14 (167). *In vivo* b-AP15 prevents tumor progression in four different solid tumor models, including HNSCC, indicating deubiquitinating activity of UCH37/UCHL5 represents a novel therapeutic target for cancer (167).

Over the last decade, the high-risk HPV infection in HNC plays a critical role in staging and prognosis, which promotes personalized therapy and the de-intensification of currently established treatment protocols based on HPV status (168). The underlying mechanisms of UCHs family in HPV-related carcinogenesis remains an enigma. It is worth mentioning that UCHL1 was specifically up-regulated by high-risk HPV in primary keratinocytes to escape innate immunity. Therefore, the precious functions of UCHL1 and other UCHs family members in HPV-related HNSCC need to be disclosed. One of the current therapeutic challenges is to find more suitable biomarkers or surrogate markers for the identity and selection of subpopulation, which would benefit from personalized and therapy. Response rates of HNSCC patients to cetuximab, the only FDA-approved molecularly target-EGFR monoclonal antibody, are only 10% (169). UCHs members have been described to interact with EGFR (170), suggesting the potential of combination therapy with UCHs members for cetuximab treatment in HNSCC.

HNSCC, like other human malignancies, is an immunosuppressive disease. Therefore, immunomodulatory treatment to overcome immune suppressive phenotypes in HNSCC patients has emerged as novel and effective strategies, which include cancer vaccines (e.g., HPV vaccines, tumor peptide antigens), cytokines (e.g., IL2, IFNγ, TNFα), specific monoclonal antibodies (e.g., anti-PD1/PD-L1, CTLA-4 antibodies) (171). Over the past 10 years, the most remarkable therapeutic advances have been achieved in immune checkpoint blockade in HNSCC. FDA approved several target immune checkpoint agents for the treatment of patients with HNSCC. However, the patients revealed different responses to these agents, with only less than 20% of the responder (172, 173). There are many challenges for the immunotherapy of HNC in the future, such as the selection of responding patients, integration into the spectrum of conventional treatment, reduction of immunosuppression in non-responding patients (174). The DUBs are involved in the regulation of innate and adaptive immune response, which sheds light on the immunoregulatory of UCHs family for combination immunotherapy in HNSCC (114, 175). In addition, a variety of patented compounds targeting UCHs members have been developed, which would prepare a path toward the outstanding achievement of genuinely personalized medicine for the treatment of cancers (176–179).

## Conclusion and Perspective

In summary, an increasing number of studies suggest that members of UCHs family exert distinct functions in a variety of human malignancies. However, available studies on UCHs in head and neck cancer are limited. It is an exciting time for HNSCC research based on the comprehensive genomic data, as the molecular landscape and altered signaling pathways has been synthetically described. But there are no genetic and proteomic screening tests routinely incorporated into the HNSCC clinically. Emerging evidence has revealed the members of UCHs are associated with the pathogenesis and clinical prognosis of HNSCC, which highlights the prognostic and therapeutic implications of UCHs for patients with HNC. Based on the available data, we have launched a joint project on the expression and function of UCHs in HNSCC, which aims to provide more evidence that UCHs might be the novel prognostic marker and therapeutic target. There are some emerging unresolved issues in HNSCC, such as: what are the precise substrates and regulators of the UCHs family? What are genetic or epigenetic events, and signaling pathways relevant to the UCHs family? Are UCHs family members able to serve as biomarkers for identifying a subset of patients to receive the optimal treatment? Can the agents targeting UCHs family become one of the novel treatment regimens? Optimization of combination regimens of immune checkpoint inhibitors and the agents targeting UCHs family may be a remarkable challenge for immunotherapy of HNSCC. Finally, whether and how the UCHs family members can be translated into the clinical management of HNC remains a formidable mission for the future.

## Author Contributions

Conception and design: CR, RZ, and JH. Writing—original draft preparation: CR and RZ. Review of the literature: CR, RZ, and SW. Project supervision: DS and S-LW. Critical revision of the manuscript: JH. Revise and resubmit: CR. All authors contributed to the article and approved the submitted version.

## Funding

CR and SW are supported by the Natural Science Foundation of the Jiangsu Higher Education Institutions of China (No. 20KJB310014, No. 18KJB320017), Natural Science Foundation of Jiangsu Province (No. BK20200878). RZ and DS are supported by Wu Jieping Medical Foundation Research Fund Project of Chinese Medical Association (No. 320.6750.19090-19). S-LW is supported by National Key R&D Program of China (Grant No. 2016YFC1303800). This study was also supported by the Priority Academic Program Development of Jiangsu Higher Education Institutions (PAPD).

## Conflict of Interest

The authors declare that the research was conducted in the absence of any commercial or financial relationships that could be construed as a potential conflict of interest.

## Abbreviations

MDM2, murine double minute 2; NOX4, NADPH oxidase 4; EGFR, Epidermal growth factor receptor; HIF-1, Hypoxia-inducible factor 1; PHLPP1, PH Domain and Leucine Rich Repeat Protein Phosphatase 1; MITF, Melanocyte Inducing Transcription Factor; CTTN, Cortactin; mTORC1, mTOR complex 1; eIF4F, Eukaryotic initiation factor 4F; TRAF2, TNF Receptor Associated Factor 2; PRDX1, Peroxiredoxin 1; SNRPF, Small Nuclear Ribonucleoprotein Polypeptide F; Smad2, SMAD Family Member 2; GRP78, 78-kDa glucose-regulated protein; E2F1, E2F Transcription Factor 1; HCF-1, Host cell factor C1; ASXL1/2, ASXL Transcriptional Regulator 1/2; MCRS1, Microspherule Protein 1; IP3R3, type-3 inositol-1,4,5-trisphosphate-receptor; ATF3, Activating Transcription Factor 3; 14-3-3 protein; SLC7A11, Solute Carrier Family 7 Member 11.
